# Role of mir-15a/16-1 in early B cell development in a mouse model of chronic lymphocytic leukemia

**DOI:** 10.18632/oncotarget.11290

**Published:** 2016-08-14

**Authors:** Chingiz Underbayev, Siddha Kasar, William Ruezinsky, Heba Degheidy, Joel Solomon Schneider, Gerald Marti, Steven R. Bauer, Diego Fraidenraich, Marilyn M. Lightfoote, Vijay Parashar, Elizabeth Raveche, Mona Batish

**Affiliations:** ^1^ New Jersey Medical School, Rutgers University, Newark, NJ, USA; ^2^ OSEL/CDRH/FDA, Silver Spring, MD, USA; ^3^ CBER/FDA Silver Spring, MD, USA; ^4^ Solid Biosciences LLC, Cambridge, MA, USA; ^5^ Rutgers School of Dental Medicine, Rutgers University, Newark, NJ, USA; ^6^ NHLBI, NIH, Bethesda, MD, USA; ^7^ Faculty of Medicine, Mansoura University, Egypt

**Keywords:** chronic lymphocytic leukemia, microRNAs, B1 progenitors, induced pluripotent stem cells, cancer stem cells

## Abstract

In both human chronic lymphocytic leukemia (CLL) and the New Zealand Black (NZB) murine model of CLL, decreased levels of microRNAs miR-15a/16 play an important role in the disease. Here we investigate the effects of this microRNA on early steps of B cell development and the capacity of miR-15a-deficient hematopoietic stem cells (HSC) and B1 progenitor cells (B1P) to reproduce CLL-like phenotype both *in vitro* and *in vivo.* Our results demonstrate that both miR-15a deficient HSC and B1P cells are capable of repopulating irradiated recipients and produce higher numbers of B1 cells than sources with normal miR-15a/16 levels. Furthermore, induced pluripotent stem (iPS) cells derived for the first time from NZB mice, provided insights into the B cell differentiation roadblock inherent in this strain. In addition, exogenously delivered miR-15a into the NZB derived B cell line provided valuable clues into novel targets such as Mmp10 and Mt2. Our data supports the hypothesis that miR-15a/16 deficient stem cells and B1Ps experience a maturation blockage, which contributes to B1 cells bias in development. This work will help understand the role of miR-15a in early events of CLL and points to B1P cells as potential cells of origin for this incurable disease.

## INTRODUCTION

CLL is the most common blood malignancy in the Western hemisphere associated with accumulation of malignant CD5^+^CD19^+^CD20^dull^CD23^+^IgM^dull^ B cells in peripheral organs [1, 2]. In the indolent form of CLL, the most frequent abnormality is a decreased expression of microRNA miR-15a/16-1 [3] from the host Dleu2 gene located in the frequently deleted 13q14 region. Deletion of this region in mice (MDR^−/−^) has been shown to lead to the accumulation of CD5^+^B220^dull^ B1 cells in peritoneal cavities (PerC) by 12 months of age in a B cell autonomous manner [4]. Importantly, mir-15a/16-1 locus deletion (not whole MDR) leads to a less aggressive CLL course [4]. Similar to human CLL and a variety of transgenic mouse models, the *de novo* animal model, New Zealand Black mouse strain, is characterized by age-associated CLL-like symptoms such as splenomegaly and CD5^+^ B1 cell hyper-proliferation with aberrant expression of Pax5, Bcl-2 and Cyclin-D1 among others [5]. We have previously discovered a point mutation and deletion in the 3′ flanking region of the mir-15a/16-1 locus in NZB mouse which are also found in some CLL patients. MicroRNAs are short 22nt long non-coding RNA molecules that are known to regulate gene expression via transcriptional repression or rarely activation [6]. The microRNA processing pathway is a multistep process which starts with RNA-pol II mediated transcription of primary transcript (pri-miR) followed by its cleavage by an enzymatic complex Drosha [7] which results in a precursor pre-miR molecule. This then is being transported to a cytoplasm by Exportin 5 protein and cleaved into a mature micro-RNA molecule by Dicer enzyme [8]. Recently, we have demonstrated that mir-15a mutation and deletion in NZB mouse are responsible for its decreased expression levels and this is due to a blockage of Drosha-mediated cleavage of primary transcript [9].

Mouse B-cell development is a complex multistep process that results in two major B populations termed B-1 and B-2 cells. The B1 population is known to play a role in innate immunity [10, 11], whereas B-2 cells that represent a major pool of B-cells, are considered as mediators of the adaptive immune response [12, 13]. Dysregulated function of B1 cells leads to the development of various autoimmune disorders [14]. On the other hand, the inherent self-renewal capacity of B1 cells confers a distinct advantage to these cells in the development of malignancies such as CLL. The origins of the two B-cells populations have been a subject of controversy between a “selection model” which advocated for the role of an antigen in B1 *versus* B2 decision making and a “layered immune system hypothesis” proposing that B1 and B2 cells are derived from two distinct progenitors which have emerged at different times during development [15–17]. The strongest evidence to support the layered model was the identification of a distinct B1 progenitor population with a Lin^−^CD45R^lo-neg^CD19^+^AA4.1^+^ phenotype [18]. However, the role of B1Ps and other lymphoid precursors has not been previously investigated in the context of CLL.

To fill in this gap in our understanding regarding the role of B1Ps in CLL, we utilized lymphoid precursors or pluripotent stem cells from the murine mouse model of CLL New Zealand Black (NZB) strain and newly generated DBA^−/−^ congenic mice (both of which have decreased miR-15a expression due to the presence of mutations in those loci) to ask the question if they can reproduce CLL-like phenotype (splenomegaly and increased B1 percentage in the spleen) both *in vitro* and *in vivo*. We hypothesized that an increase in B-1 lineage commitment leads to the eventual emergence of a malignant B-1 clone and concurrent CLL development. Our work uncovers previously unknown early miR-15a deficiency associated developmental events in a mouse model of CLL and provides evidence for the role of B1 progenitors as a novel potential source population for origin of CLL.

## RESULTS

### DBA^−/−^ congenic mice exhibit B cell maturation defects accompanied by B1 and T cells expansion

**Figure 1:**
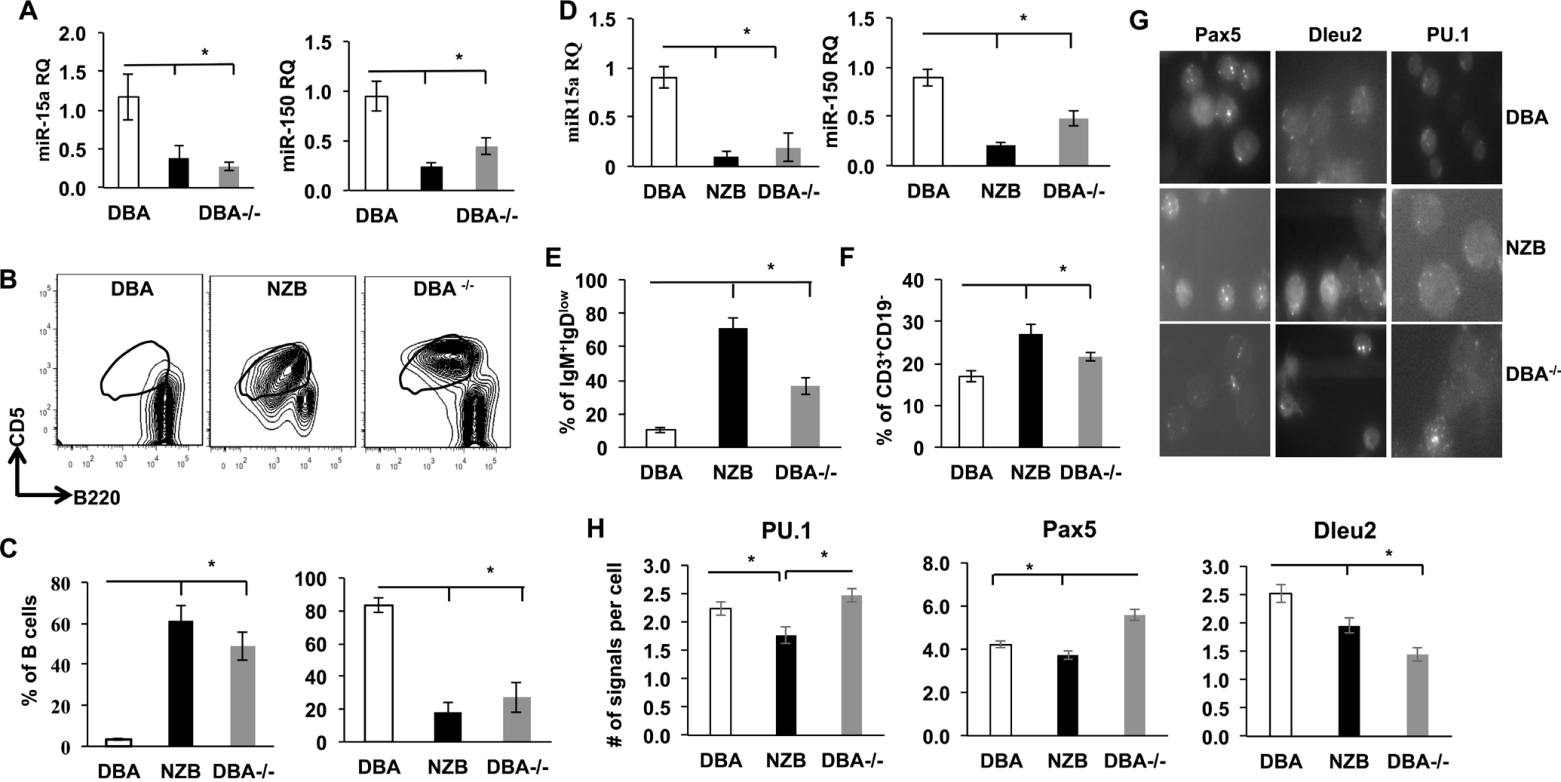
Comparison of splenic phenotype in control DBA, NZB and DBA congenic (DBA^−/−^) mice **A.** TaqMan real-time PCR quantification of miR-15a and miR-150 levels in sorted B1a subpopulations from spleen; *N* = 3, columns represent mean RQs, bars are SEMs; **B.** Flow cytometry staining for B1 CD5+, B220^dull^ cells (gated on CD3-CD19+). This population was sorted and used for analysis in panels C and D. **C.** Quantification of B1 (left) and B2 (right) cells in DBA, NZB and DBA−/− congenic mice spleen; *n* = 3. **D.** TaqMan PCR levels of miR-15a and miR-150 in sorted B2 subpopulation from spleen; RQ is relative quantification normalized to snRNA U6 expression; **E.** Flow cytometry quantitative analysis of immature IgM+IgDlow B cells; columns are means, bars are SEMs; n≥3. **F.** Flow cytometry quantitative analysis of CD19-CD3+ T cells in spleen, age = 12 months; columns are means, bars are SEMs; n≥3. **G.** Representative pictures of single molecule RNA-FISH with Pax5, Dleu2 and PU.1 probes; **H.** Quantitative analysis of Pax5, PU.1 and Dleu2 transcripts in splenocytes derived from DBA, NZB and DBA−/− mice by RNA-FISH (only Pax5+ B cells were counted). At least 50 individual cells from each animal were counted from at least 3 (*n* = 3) animals of the same strain and analyzed. Columns represent average mRNA molecules per cell and bars are SEMs. Asterisks represent statistical difference (*p* < 0.05).

In order to study the effects of the miR-15a/16-1 deficiency on the mouse B cell development the congenic mice were generated by consecutive crossbreeding wild type DBA and CLL mouse model NZB mice with mutant mir-15a/16-1 selection. The resultant strain termed DBA^−/−^ was used for aging followed by further analysis [9]. First, we found that the level of miR-15a was decreased in both B1 and B2 cells derived from NZB and DBA congenics (DBA^−/−^) when compared to the wild-type DBA (Figure 1A), which positively correlated with Dleu2 levels in splenocytes. To test the effect of decreased miR-15a/16 on B lineage commitment /maturation *in vivo*, the B cell phenotype of the aged DBA^−/−^ congenic (mutated mir-15a/16-1) animals was compared to control DBA (with normal miR-15a) and NZB (reduced miR-15a). The DBA^−/−^ strain demonstrated increased spleen sizes relative to the parental age matched DBA strain, but not to the extent seen in the NZB animals (Figure S1). Further analysis of the types of cells contributing to the enlarged spleen indicated that an increase in the percentage of B-1 cells was associated with splenomegaly (Figure 1B). The percentage of B-1 cells was increased in DBA congenic mice with mutated *mir-15a/16* loci whereas the numbers of conventional B2 cells decreased relative to the DBA wild-type control spleens (Figure 1C).

Another microRNA, miR-150, which is known to be expressed specifically in mature lymphocytes but not their progenitors [19] was also expressed at significantly lower levels in NZB and DBA^−/−^ B cell sub-populations, which may be due to their incomplete maturation (Figure 1D). Indeed, in the NZB strain, B1 cells also exhibit diminished IgD expression, indicating their immature status. This feature has not been yet directly linked to miR-15a defects. Our data shows that in both NZB and DBA^−/−^ congenic mice the size of immature IgM^+^IgD^low^ B cell populations are higher than those observed in the DBA spleen (Figure 1E) suggesting a defect in conventional B cell maturation.

T cell abnormalities have been previously reported to play a substantial role in human CLL pathogenesis [20]. Analysis of NZB and DBA^−/−^ spleens revealed a significantly higher percentage of CD3^+^CD19^−^ T cells (Figure 1F). Furthermore, RNA-FISH analysis of PU.1 levels which has been previously shown to abrogate B2 cell development in favor of the B1 lineage [21] showed its downregualtion in NZB but not DBA^−/−^ spleens (Figure 1G and 1H). This could reflect the fact that at this stage of development miR-15a deficiency alone is not capable of affecting PU.1 levels in congenic mice splenocytes. Whereas Pax5, which is a master B cell regulatory gene known to be dysregulated in NZB mouse model, appeared to be aberrantly overexpressed in DBA^−/−^ spleens only (Figure 1H) suggesting a more intricate regulation between miR-15a defect and this transcript levels.

Collectively, our data shows that miR-15a deficiency alone can affect B cell maturation and lead to accumulation of B1 and T cells at the expense of B2 cells compartment *in vivo*.

### B cell progenitors' abnormalities

**Figure 2:**
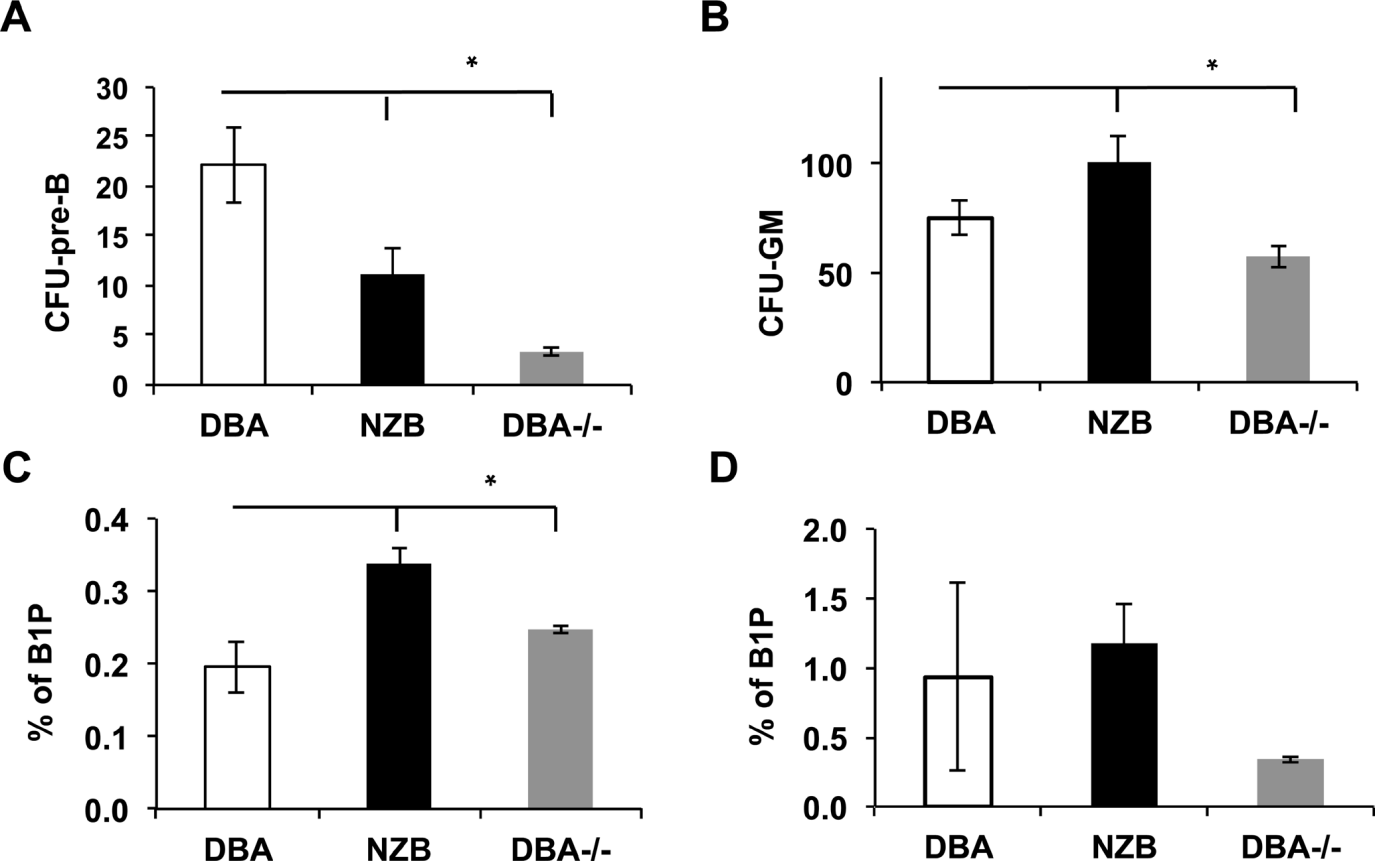
B1 progenitors (B1P) and colony forming units (CFU) analysis **A.** Pre-B CFU assay for bone marrow cells derived from the indicated mouse strains; **B.** CFU-GM assay of bone marrow cells derived from the indicated mouse strains. Columns are means and bars are SEMs. Asterisks represent statistical difference (*p* < 0.05). **C.** Flow cytometry quantitative analysis of B1 progenitors in the spleen of the indicated mouse strains (age = 12 months, *n* = 3); columns represent means and bars are SEMs. **D.** Flow cytometry quantitative analysis of B1 progenitors in the bone marrow of the indicated mouse strains (age = 12 months, *n* = 3); no statistical difference observed. B1 progenitors were defined as Lin^−^AA4.1^+^CD19^+^B220^neg/dull^ population.

**Figure 3:**
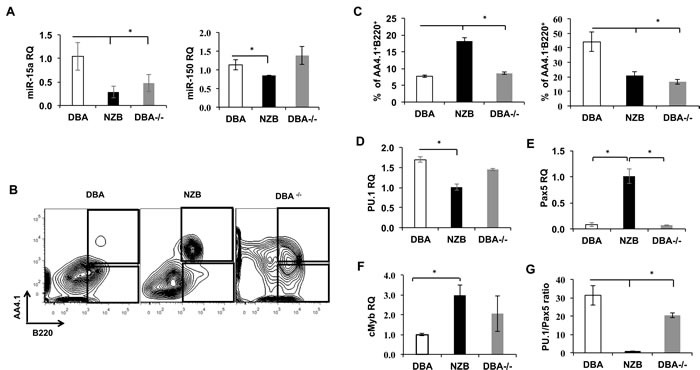
*In vitro* differentiation of bone marrow progenitors from miR-15a-deficient mice **A.** The levels of miR-15a and miR-150 in LSK cells co cultured with OP9 stromal cells at day 11 measured by TaqMan qPCR; **B.** Representative flow cytometry analysis of DBA, NZB and DBA−/− LSK derived immature (top box) and mature (bottom box) B cell progenitors at day 11 of OP9 co-culture. **C.** Quantitative flow cytometry analysis of mature AA4.1^−^B220^+^ (right) and immature (left) B cells progenitors. *N* = 3, columns represent means and bars are SEMs; Transcript expression analysis of **D.** PU.1, **E.** Pax5, and **F.** cMyb measured by qPCR assay are shown. MicroRNA levels were assessed using TaqMan assays with custom probes for has-miR-15a and has-miR-150. Protein-coding genes transcripts measurements were done by qPCR with the use of SYBR green master mix and specific primers; *n* = 3, columns represent means and bars are SEMs; **G.** The ratio of PU.1 to Pax5 gene expression in LSK cells at day 11 of co culture with OP9 cells. Asterisks represent statistically significant difference (*p* < 0.05).

Observed maturation defects prompted us to look into B cell progenitors' compartment of DBA^−/−^ animals. First, the pre-B colony forming units (CFU-pre-B) assay revealed a diminished capacity of the NZB and DBA^−/−^ bone marrow cells to give rise to pre-B colonies *in vitro* (Figure 2A) but not to CFU-GM counts (Figure 2B) suggesting the B lineage specific effect. The B1P population analysis revealed their elevated percentage in the spleens (Figure 2C) but not in the bone marrow (Figure 2D) of age-matched NZB and DBA^−/−^ congenic mice. Furthermore, no difference in B1P levels was observed in younger animals suggesting that this is an age-associated phenomenon (data not shown). Recently, we have shown that DBA congenic animals exhibit an increased percentage of lineage^−^sca1^+^c-kit^+^ (LSK) cells in DBA^−/−^ congenic mice [9]. To test their ability to give rise to B lineage cells, LSK progenitors derived from the DBA, NZB and DBA^−/−^ mice bone marrow were differentiated *in vitro* under conditions favoring B cell development. Both NZB and DBA^−/−^ sources showed decreased levels of miR-15a (Figure 3A) and produced more immature AA4.1^+^B220^+^ early B cell progenitors relative to DBA LSKs (Figure 3B and 3C). Furthermore, NZB-LSK derived cells harvested on day 11 had reduced miR-150 levels (Figure 3A, right panel) consistent with the significantly increased immature B cells observed during analysis of surface markers. However, the DBA^−/−^ congenic mice did not have decreased miR-150 relative to the control wild-type DBA strain. This highlights that additional genetic modifications other than decreased miR-15a/16 are responsible for the abnormalities observed in the CLL strain, NZB. The analysis of the mature AA4.1^−^B220^+^ population revealed that NZB and DBA^−/−^ sources produced less mature cells as compared to the wild-type DBA LSK source of cells (Figure 3C right panel). The same pattern has been observed at later stages of co-culture system (data not shown).

Quantitative PCR analysis showed that NZB LSK but not DBA^−/−^ have decreased expression of PU.1 (Figure 3D), and the expression of Pax5 was found to be significantly increased only in the NZB sources of LSK cells (Figure 3E). In addition, the levels of cMyb, which is a direct target of miR-15a, were significantly higher in NZB but not DBA^−/−^ when compared to DBA LSK at day 11 of co-culture (Figure 3F). Since Pax5 is known to be overexpressed in NZB CLL cells and PU.1 KO was previously shown to result in B1 cells accumulation, we reasoned that the ratio of PU.1 to Pax5 might reflect the bias towards the conventional B2 development. To this end our results show that this ratio was significantly lower in both NZB and DBA^−/−^ LSK cells at day 11 of co-culture (Figure 3G) suggesting a possible B1 cell development program domination at this stage of maturation.

Overall, even though the DBA^−/−^ LSK phenotypical profile appeared to be similar but not identical to NZB counterpart due to other factors present in the latter, our data indicate that miR-15a/16-1 deficiency alone can lead to maturation abnormalities in the course of early B cell development *in vitro*.

### DBA^−/−^ HSCs and B1Ps produce B1 cells in NSG recipients

**Figure 4:**
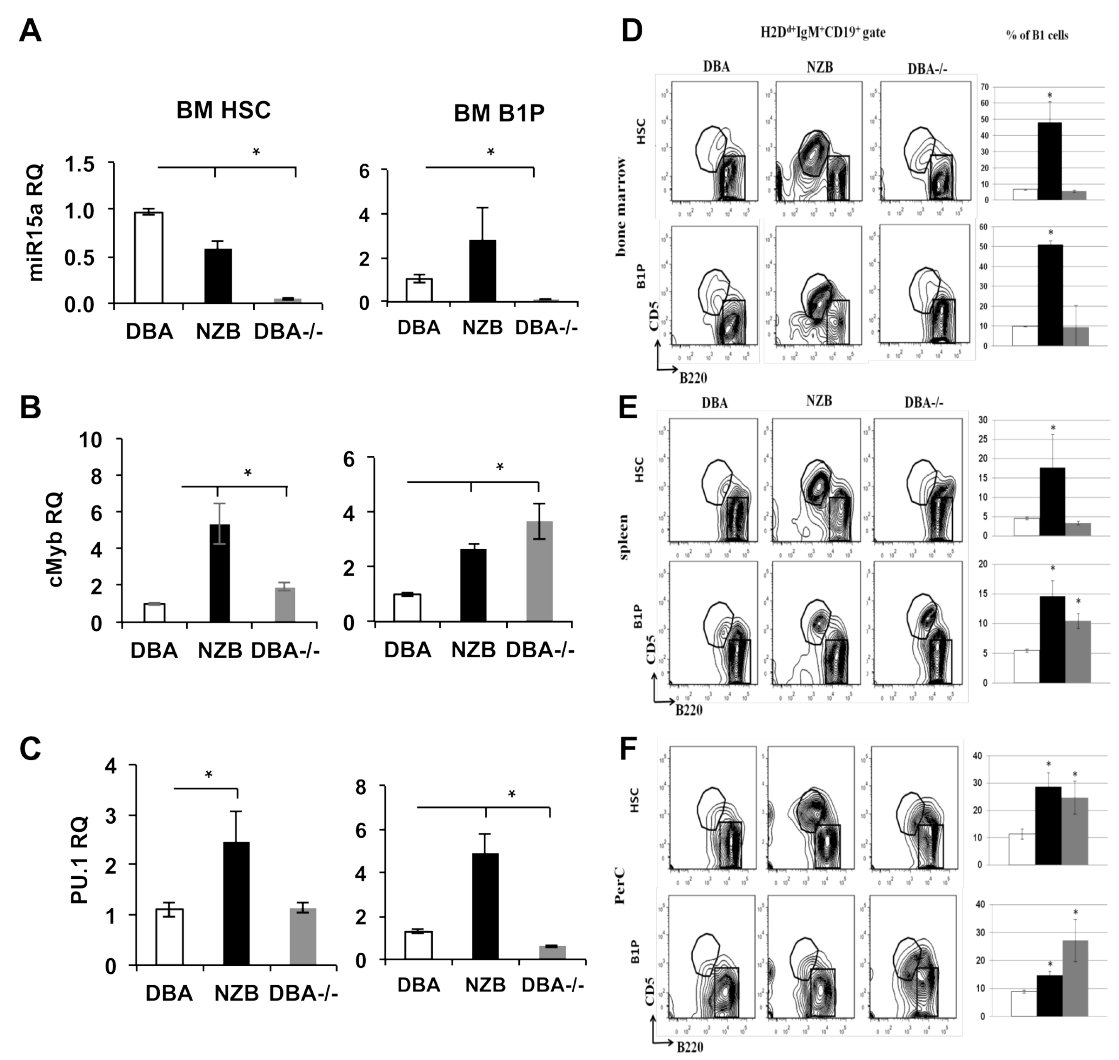
Repopulation potential of DBA, NZB and DBA^**−/−**^ congenic mice derived HSC and B1 progenitor cells in immunodeficient NSG recipients at 48 days post injection **A.** Mir-15a, **B.** cMyb and **C.** PU.1 expression levels in DBA, NZB and DBA^−/−^ HSC and B1Ps. MicroRNA levels were assessed using TaqMan assays with custom probes for hsa-miR-15a. Quantitative PCR for protein coding genes was performed with the use of SYBR green master mix and specific primers; *n* = 3, columns represent means and bars are SEMs. Flow cytometry analysis of **D.** bone marrow, **E.** spleen and **F.** peritoneal cavity cells from NSG recipients. Recipient NSG mice were analyzed 48 days post injection for the presence of donor cells (H2D^d+^) which also expressed markers for B1 progenitors as a percentage of the total donor cell population in the bone marrow and spleen (mean ± SEM, *n* ≥ 3, *p* < 0.05). Cells were gated on donor H2D^d+^IgM^+^CD19^+^ mature B cells and analyzed for CD5 and B220 expression. The B1 population (CD5^+^B220^dull^) is indicated in the circle and the B2 population (CD5^−^B220^hi^) is indicated in the box. Quantitative analysis of B1 cells is shown as mean ± SEM on the right side of each panel. Open bars represent DBA, black bars are NZB and grey bars are DBA^−/−^ source. Asterisks indicate significant difference from control DBA donors.

To further test if mir-15a mutation alone can contribute to B1 cell expansion, the HSCs and B1P cells derived from the bone marrow of DBA, NZB and DBA^−/−^ congenic mice were transferred into sub-lethally irradiated immunodeficient NSG recipients. Before the adoptive transfer, the analysis of HSC sources showed significantly decreased miR-15a expression levels in both NZB and DBA^−/−^ sources (Figure 4A, left panel), whereas B1P cells demonstrated significantly decreased miR-15a only in the DBA^−/−^ congenic sources (Figure 4A, right panel). Expectedly, cMyb, a known target of miR-15a, was up-regulated in HSC and B1P sources with decreased miR-15a (Figure 4B). PU.1 levels on the other hand showed a larger variation between cell sources possibly reflecting the heterogeneity of sorted populations (Figure 4C).

Flow cytometry analysis at 48 days post-injection showed that B2 lineage was the predominant B cell subpopulation when cells from the wild-type DBA strain were transferred. The B2 cell production from B1Ps may reflect the impurity of cells sorting. However, there were differences between NZB and DBA^−/−^ donor progenitors' derived cells compared to the wild-type DBA counterparts. Both the HSC and B1P cells derived from NZB donors led to CD5+B220+ B1a cells overexpansion in all peripheral sites of the recipients (Figure 4D-4F).

Strikingly, only B1P cells but not HSC derived from DBA^−/−^ mice bone marrow repopulated the recipients' spleen more efficiently than their DBA counterparts (Figure 4E), whereas both HSC and B1P from DBA^−/−^ donors led to B1a cells expansion in recipients' peritoneal cavities. This was further confirmed by histology staining (Figure S2A-C). In follicles, B1 cells were found predominantly in the marginal zones and reduced in the germinal centers. The recipients of the wild-type stem cells had well-formed germinal centers, whereas recipients of NZB stem cells had increased marginal zone regions consistent with a B1 expansion and reduced B2 repopulation. The DBA^−/−^ congenics were similar to the NZB in the follicle morphology (Figure S2B-C). The NSG recipients also demonstrated splenomegaly related to the source of stem cells. The donor NZB HSCs resulted in the most dramatic splenomegaly (Figure S2A) but DBA^−/−^ B1P injected recipients developed slightly bigger spleens (by mass) than their DBA counterparts (not statistically significant). No significant differences in B2 cells counts have been observed (Figure S3A). Additionally, donor HSCs from NZB and DBA^−/−^ bone marrow produced significantly more CD11b^+^ cells in the bone marrow of recipients (Figure S3B) and T cells in the spleens of NSG recipients (Figure S3C). Similarly, the B1Ps from NZB and DBA^−/−^ donors resulted in a noticeable increase in CD11b^+^ cells in the recipient spleens (Figure S3B). These results show that HSCs and B1Ps with mir-15a defect alone can reproduce CLL like phenotype *in vivo*.

### NZB pluripotent stem cells have a defect in B cell differentiation

Since NZB embryonic stem (ES) cells are not able to contribute to germline transmission [22] we employed both NZB ES and newly generated NZB iPS cells to study early events in stem cell commitment to B lineage. We used NZB splenic stromal fibroblasts (NSF) to establish iPS cell lines for CLL mouse model for the first time. This was done by means of lentiviral transduction of pluripotency factors OCT4, SOX2 and KLF4 followed by culture maintenance in ES media with differentiation inhibitors (Supplemental Methods). Pluripotency was confirmed by alkaline phosphatase assay (Figure S4A), transcript expression measurements of murine Oct4, Sox2, Klf4, cMyc and Nanog genes (Figure S4B), and immunofluorescence staining for SSEA1 and Nanog (Figure S4C). Additionally, we quantified Nanog expression levels to compare pluripotency potential of the established cell lines (Figure S4E). Finally, NZB iPS cells were tested in teratoma formation assays and found to be able to give rise to tissues of all three germ layers (Figure S4D and S4G).

**Figure 5:**
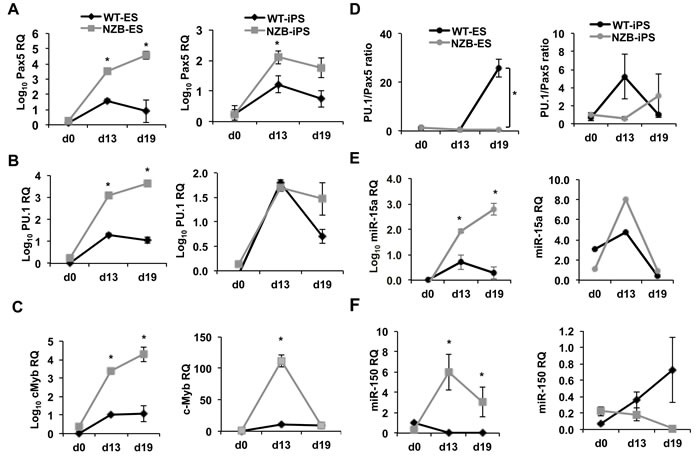
Quantitative PCR analysis of *in vitro* differentiated NZB ES/iPS cells The expression levels of **A.** Pax5, **B.** PU.1, **C.** cMyb, **D.** miR-15a, **E.** miR-150 in ES (left panels) and iPS (right panels) cells during *in vitro* differentiation. **F.** The calculated ratios of PU.1 to Pax5 expression in ES (left) and iPS (right) cells. Relative quantification (RQ) and log10 RQ are indicated on the axes; *n* = 3, *p* < 0.05 is considered significantly different from the previous time point and marked with an asterisk. ES and iPS cells were pre-differentiated as embryoid bodies for 7 days and plated in semisolid pre-B methocult medium for 12 days. qPCR assays were performed using SYBR green master mix and custom primers for protein coding gene transcripts. First strand cDNA synthesis was performed by using SuperScript First-Strand Synthesis System (Invitrogen) using oligo-d(T)n. MicroRNA measurements were done using TaqMan assays for mmu-miR-15a and mmu-miR-150. TaqMan assay for U6 was used as a housekeeping control.

To test their differentiation capacities both NZB ES and iPS cells were differentiated in pre-B methocult medium (Figure S4F) followed by qPCR analysis of key B cell factors as well as miR-15a and miR-150 measurements. The levels of Pax5 and PU.1 increased from day 0 in the presence of IL-7 and Flt3L in all stem cell sources (Figure 5A and 5B). However, there were differences between the types of iPS cells. ES cells induced to differentiate into B cells demonstrated much higher levels of these transcription factors compared to iPS cells (Figure 5A-5C, left *versus* right panels). This may reflect the fact that iPS cells are artificially induced and can still retain a residual OSK transgene expression, which would have an inhibitory effect on differentiation program. Strikingly, NZB ES cells showed higher expression of Pax5, PU.1 and cMyb transcripts than the wild type ES cells (Figure 5A-5C) suggesting a bias towards an initial commitment to B lineage within the NZB background. Similar pattern has been observed for iPS cells with an exception of PU.1 expression (Figure 5B, right). Nevertheless, at day 19, the PU.1/Pax5 ratio was lower in NZB ES cells suggesting that they experience PU.1 deficiency relative to Pax5 expression (Figure 5D). In addition, the levels of miR-15a in differentiated NZB ES cells followed the expression of Pax5, PU.1 and cMyb transcripts (Figure 5E), which may reflect an enhanced B cell differentiation of NZB lineage cells at this early stage. This was further supported by an elevated expression of miR-150 in NZB ES cells (Figure 5F), which was used as a maturation marker in this context. No significant differences in miR levels were observed in iPS cells.

To test the capability of iPS/ES cells to generate mature B cells *in vitro*, iPS and ES derived embryoid bodies (EB) cells were co-cultured with OP9 stromal cells in media supplemented with B cell differentiation cytokines. In this system, NZB ES- or iPS-derived EB cells failed to generate any B cell lineage cells in the settings used. Only wild-type ES cells produced CD19^+^ and B220^+^ cells in the OP9 system (Figure S5) whereas NZB ES or iPS cells did not survive past 2 weeks of differentiation (data not shown).

This data shows that NZB background favors the earliest steps of B cell development but poses a roadblock for further differentiation.

### Effects of exogenous miR-15a/16 on mature B1 cells

**Figure 6:**
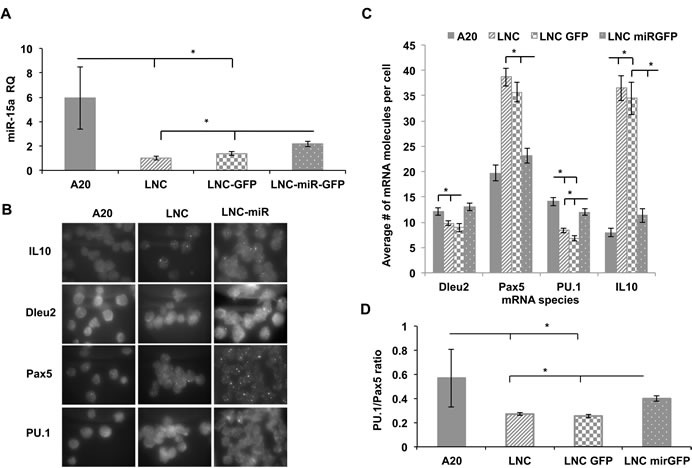
Gene expression analysis of NZB B cell line (LNC) transduced with miR-15a lentiviral construct Four cell lines including the non-NZB B cell line A20, the NZB cell line, LNC and two sublines of LNC which had been transduced with a lentivirus containing GFP (LNC-GFP) or LNC transduced with a lentivirus containing both GFP and miR-15a/16 (LNC-miR-GFP) were studied:. **A.** Quantitative PCR TaqMan assay for mir-15a levels in LNC cells lentivirally transduced with wild type mir-15a construct in comparison to non-transduced and non-NZB cells; **B.** Representative RNA FISH microscopy images of individual transcripts. The slides were imaged using probes specific to Pax5, PU.1, Dleu2 and IL-10 RNA molecules; **C.** Quantitative analysis of RNA-FISH data to obtain average number of specific mRNAs per cell. At least 100 cells were analyzed; **D.** Calculated PU.1 to Pax5 expression levels ratio. Each cell was simultaneously probed for both PU.1 and Pax5 and the ratio was determined; *n* = 3, asterisks represent statistically significant difference (*p* < 0.05).

Since many of miR-15a target genes are expressed in early B lineage cells, we employed NZB cell line (LNC) to uncover potentail additional targets which may also play a role in stem cell commitment to the B1 lineage. LNC cell transduction with a construct bearing a wild type murine miR-15a/16 locus resulted in a significant increase in mir-15a levels (Figure 6A). We had previously found that higher levels of miR-15a in LNC cells result in decreased viability and proliferation [23]. This suggests that miR-15a targets genes that are critical for the survival of the malignant B1 cells. To investigate the spectrum of genes affected by miR-15a, we first performed a single molecule RNA-FISH analysis of several critical transcripts and showed that forced miR-15a expression in malignant B1 cells could suppress otherwise overly up-regulated target mRNAs such as Pax5 and IL10 (Figure 6B and 6C). In contrast, transcripts that are decreased in malignant CLL cells, such as Dleu2 and PU.1, were found to be up-regulated after exogenous overexpression of miR-15a/16 (Figure 6C). The PU.1 up-regulation was also confirmed at the protein level (Figure S6A). Notably, the PU.1/Pax5 ratio that we used as a putative B2/B1 fate predictor above has been found to be significantly elevated in LNC-miR-GFP cells as compared to the LNC-GFP cells (Figure 6D).

Additionally, RNA-Seq analysis revealed a number of differentially expressed genes in LNC cells including IL-10 and Nfkb1, which are known to be critical in human CLL pathogenesis (Figure S6B). Surprisingly, only two transcripts showed a significant change of expression levels in LNC cells with exogenously overexpressed miR-15a. One of them was metalloprotease 10 (Mmp10), which is an important gene involved in lymphoid growth [24] and metastasis [25]. Mmp10 transcript was dramatically suppressed in LNCs expressing exogenous miR-15a (Figure S6C). This was further validated by Western blot analysis (Figure S6A). On the other hand, miR-15a overexpression led to a significant increase in Mt2 gene expression (Figure S6C). Mt2 belongs to the family of metallothioneins that have been previously shown to inhibit the immunosuppressive type 1 regulatory T (Tr1) cells producing IL10 [26].

Collectively, our data shows that miR-15a has a negative effect on critical CLL-associated genes such as IL10 and Pax5. It can also restore PU.1 expression at both mRNA and protein levels. In addition, we found two novel potential targets Mmp10 and Mt2 that are differentially affected by miR-15a expression.

## DISCUSSION

New Zealand Black strain is the only *de novo* CLL mouse model that is not derived using transgenic methods. It possesses several genetic defects other than mir-15a/16-1 mutation hence it is not an ideal model to study effects of individual mutations. Recently, we reported generation of a DBA congenic mouse model with an NZB-derived mutated mir-15a/16 locus, and showed that this mutation is responsible for reduced levels of mature miR-15a due to the defects in microRNA processing [9]. Several approaches have been exploited in this work to further understand the role of miR-15a in B cell development using this mouse model coupled with both *in vitro* and *in vivo* assays.

First of all, we have been able to confirm that miR-15a deficiency alone is able to alter the mouse phenotype that has manifested in overproduction of B1 and T cells at the expense of conventional B2 cells. This was accompanied by an accumulation of IgM^+^IgD^low^ population and low miR-150 expression in the spleens of congenic animals suggesting a maturation deficiency. The fact that DBA^−/−^ congenic bone marrow contains significantly less pre-B CFUs can be explained by at least two mechanisms in which decreased miR-15a/16 might mediate this effect. First, it could reflect an early B cell development defect in bone marrow progenitors, or secondly, it can be attributed to an enhanced migration of B cell progenitors out of bone marrow. The first possibility is supported by our data on diminished maturation capacity observed in miR-15a deficient mouse strains both *in vitro* (Figure 3B and 3C) and *in vivo* (Figure 1D and 1E). The second scenario is yet to be elucidated, however, previous work by others suggests that PU.1 deficient AA4.1^+^ B cell progenitors experience homing issues when injected into syngeneic recipients [27]. This mechanism would offer migratory advantage to mir-15a/PU.1-deficient progenitors and eventually lead to overexpansion of B1 cells and their progenitors in peripheral organs, which is also supported by our *in vivo* data (Figure 2C and 2D). Indeed, the results obtained from our adoptive transfer experiments have underlined the contribution of miR-15a into early fate decision making in the B cell compartment. This data implies that early events associated with point mutation in mir-15a locus alone can be partially responsible for B1 cells expansion. Although, the purified B1P cells produced a significant amount of B2 cells in the recipients reflecting their possible contamination with other progenitors, the enrichment for miR-15a-deficient DBA^−/−^ B1P cells using defined surface markers led to a higher number of B1 cells in the spleens of the recipients relative to HSC source. Given the fact that miR-15a is targeting an anti-apoptotic Bcl-2 gene [28], its deficiency in B1P progenitors might provide them with survival advantage during B cell development which is supported by their elevated percentage in the bone marrow of miR-15a-deficient congenic animals. Furthermore, our results suggest that miR-15a/16 may also indirectly regulate miR-150 whose deficiency was shown to result in an expansion of B1 cells by directly targeting c-Myb transcript [29]. In fact, both miR-150 and miR-15a/16 are confirmed suppressors of this transcription factor [30]. However, the loss of miR-150 alone does not lead to a detectable increase in cMyb protein levels in mature B1 cells [29] suggesting that another hit is required to promote B1 cells expansion through cMyb overexpression. To this end, miR-15a deficiency can actually favor B1 overexpansion by allowing high levels of cMyb with concurrently low PU.1/Pax5 expression levels ratio during early steps of B cell development (Figures 3G and 5D). Thus the balance between PU.1 and Pax5 seems to be critical for that matter and as we have shown can be restored by forced miR-15a expression (Figure 6D). The up-regulation of PU.1 following exogenous delivery of miR-15a could have been due to down-regulation of cMyb, a known suppressor of PU.1. On the other hand, PU.1 transcription factor levels are also impacted by BSAP (Pax5 gene protein), which can bind to PU.1 promoter and repress it [31]. Previously, we have shown that BSAP can modulate miR-15a levels by directly binding to Dleu2 promoter [23]. This can create an auto-regulatory loop in which low levels of miR-15a would allow suboptimal PU.1 expression affecting B2 lineage commitment program.

Additionally, RNA-seq analysis on LNC cells showed that miR15a is capable of down-regulating a number of critical genes such as IL10 and Mmp10. IL10 knockout experiments in mice have previously underlined its role in B1 cell expansion and development of CLL [32], whereas Mmp10 is potentially a novel CLL gene, which is known to be induced in lymphoma cells thereby accelerating the tumor growth [33]. Mmp10 is also an important mediator of cell migration and invasion [34] and considering our *in vivo* data on DBA^−/−^ -derived B1 progenitors' accumulation in the spleen and PerC of NSG recipients, it is reasonable to suggest that miR-15a deficient B1Ps might have a migrational advantage and stronger ability to “invade” peripheral organs. On the contrary, Mt2 gene was found to be the sole, significantly up-regulated transcript upon exogenous transduction of miR-15a. Mt2 is a member of metallothioneins (MT) family, which comprises of a set of genes known to regulate heavy metal detoxification and oxidative stress [35], and it has been previously shown that mice deficient in MTs exhibit overproduction of IL10 by Tr1 cells [26]. Further work in this direction could uncover putative links between miR-15a deficiency and MT-mediated immunomodulation in CLL.

The overproduction of CD11b^+^ myeloid lineage cells and T cells in NSG recipients requires further investigation. However, it is worth noting that both myeloid CD11b and T cell marker CD5 are expressed in malignant B1a subset in CLL. Thus miR-15a deficiency can affect their expression via cMyb which is known to play a critical role in both myeloid and T cells development [36, 37].

In addition, the NZB iPS generation was performed for the first time in a mouse model of CLL and these cells exhibited a B cell differentiation blockage when differentiated *in vitro*. Since B1 cells appear prior to B2 cells in ontogeny, short-term B cell cultures may mimic ontogeny and the early NZB B lineage cells might be of B1 origin. Furthermore, similar to LSK progenitors differentiated *in vitro* (Figure 3G), NZB ES cells exhibited a reduced PU.1/Pax5 ratio at day 19 of culture (Figure 5D) suggesting a imbalance between these two important factors. Strikingly, the use of OP9 system, which is commonly used to differentiate ES/iPS cells into B lineage cells, failed to produce any B cells from NZB ES or iPS cells under standard conditions. Only wild-type iPS and ES gave rise to B220^+^ and CD19^+^ cells *in vitro*. This may reflect a roadblock inherent in NZB-lineage pluripotent stem cells to commit to B2 lineage *in vitro*. Thus a generation of DBA^−/−^ iPS cells would provide benefits for studying an individual effect of mir-15a mutation on early lineage commitment *in vitro*. Nevertheless, NZB iPS/ES cells can offer clues to uncover roadblocks in B cell differentiation and provide valuable tools for CLL mouse modeling.

In summary, decreased miR-15a/16 levels alone have a significant effect on HSC and B1P cell differentiation with a bias towards B1 and T cell development at the expense of conventional B2 program. This has profound implications on our understanding of the development of CLL and suggests that, at least in some patients, the initial events precipitating in the development of CLL occur long before the expression of malignant clones as previously demonstrated for HSC population in human CLL [38]. Specifically, this study implicates B1P cells as a potential source population for murine CLL cell of origin. Finding a human counterpart for B1 cell progenitor subpopulation would offer significant insights into our understanding of CLL clonal evolution and its mechanisms of resistance in humans.

## MATERIALS AND METHODS

### Cell lines

The NZB malignant B-1 cell line termed LNC was derived in our lab and used as an *in vitro* model of murine CLL [39]. The BALB/C B cell lymphoma A20 cells line (ATCC^®^ TIB-208) was used as a non-NZB control. OP9 (ATCC^®^ CRL-2749) stromal cell line served as feeder layer for ES/iPS cells *in vitro* differentiation experiments. NZB ES cells were a kind gift from Dr. Ken-Ichi Yagami (Laboratory Animal Resource Center, University of Tsukuba, Tsukuba, Ibaraki Japan). Wild type ES cells were kindly provided by Dr. Diego Fraidenraich (Department of Cell Biology and Molecular Medicine, New Jersey Medical School, Rutgers University, Newark, NJ).

### Mice

NZBBlNJ, DBA2J and NSG mice were purchased from Jackson Laboratory and housed under standard pathogen-free conditions at the research animal facility at Rutgers University. DBA congenic strain (DBA^−/−^) was generated by systematic back-crossing to NZB strain following the protocol described elsewhere [32]. All studies were in compliance with principles of laboratory animal care guidelines.

### Flow cytometry and cell sorting

Flow cytometry experiments for B-1 malignant cells were performed using single-cell suspensions generated from spleen, bone marrow and peritoneal cavity (PerC) lavage from the animals. The isolated cells were surface stained with anti-mouse IgM, CD5, CD45R (B220), CD19, CD11b, CD3 and IgD antibodies. Phenotypically, B1 cells were defined as CD3^−^CD19^+^CD5^dull^B220^dull^ population whereas B2 cells were defined as CD3^−^CD19^+^CD5^−^B220^hi^ population. For adoptive transfer experiments, H2d antibody was used to discriminate between the donor and recipients NSG cells. Data was acquired on a LSR II and analyzed using FlowJo software. For a full list of antibodies see Table S1.

### Colony formation assays

Bone marrow cells derived from DBA, NZB and DBA^−/−^ mice were filtered and seeded onto semisolid methocult medium (M3630 for pre-B and M3534 for granulocyte/myeloid CFUs, Stemcell Technologies, Vancouver, Canada) at 10^5^ cells per well density following manufacturer protocols. The colonies were counted by microscopy seven to fourteen days later.

### iPS cells generation and lentiviral constructs

The NZB iPS cells were induced as previously described [40]. Briefly, the packaging and envelope plasmids along with polycistronic lentiviral construct were co-transfected into 293T cells. The pseudoviral particles were concentrated by ultracentrifugation and used to transduce NZB mouse splenic stromal cells (NSF) cells. ES-like cell colonies were individually picked 2-3 weeks later and expanded for analysis and subsequent experiments. For LNC transduction experiments, the custom miR-15a-lenti-GFP lentiviral construct and the empty lenti-GFP vector (SBI, Mountain View, CA) were used to generate pseudo-lentiviral particles by means of 293T cell transfection. The pseudoviral aliquots were added dropwise to the LNC pre-seeded (24 h) culture plates at MOI 10. The plates were incubated at 32°C for 24 hours. The transduction efficiency was tested using fluorescence microscopy followed by quantitative assessment by flow cytometry.

### Quantitative PCR

Gene expression levels were measured using Power SYBR Green Kit (Ambion Inc) or SYBR Low-ROX Kit (Bioline) and custom designed primers (Table S2) on total RNA derived cDNA templates. β-actin expression was used as a normalization housekeeping control. For microRNA measurements, the cDNA was prepared using the TaqMan MicroRNA Reverse Transcription Kit (Applied Biosystems) according to the manufacturer's protocols. The real-time quantification was performed using pre-made TaqMan Assays (Life Technologies): mmu-miR-15a (assay ID 000389) and mmu-miR-150 (assay ID002637); U6 assay was used as a normalizer control (assay ID001973).

### RNA FISH

Single-molecule RNA FISH analysis was done on Dleu2, PU.1, IL10 and Pax5 RNAs using custom probes as previously described [41]. A set of 35 probes was designed with a 3′ amino modification (Biosearch Technologies, Novato) and coupled with different fluorophores to hybridize to each target RNA. LNC cells were analyzed with separate individual probes whereas primary splenocytes were probed for three target mRNAs simultaneously to count Pax5^+^ B cells only.

### Western blotting

Cell lysates were collected in lysis buffer containing protease and phosphatase inhibitors. Equal amounts of total protein were separated on 7.5% SDS-PAGE gels, transferred to Nitrocellulose 0.45μm membrane (BIO-RAD), and subsequently probed with antibody against MMP-10, PU.1, c-Myb, BSAP and β-actin.

### Immunohistochemical staining

Formalin-fixed tissue sections of spleen from NSG mice stained with hematoxylin and eosin, and analyzed using an Olympus BX40 microscope (Olympus, Center Valley, PA, USA) with 10X and 20X objective lenses. Digital images were taken and analyzed.

### *In vitro* differentiation

Stromal OP9 feeder cells were used for iPS or ES *in vitro* differentiation following a previously published protocol with minor modifications [42]. ES or iPS cells were harvested as a single cell suspension and added to OP9 monolayer. The cytokines were supplemented at concentrations of 5 ng/mL for Flt-3L and 1 ng/mL for IL7. On day 12, the cells were re-plated on fresh OP9 with 5 ng/mL Flt-3L and 1 ng/mL IL-7 and the medium was changed every 2-3 days. The analysis was performed at intervals corresponding to the outgrowth of non-adherent cells in the co-culture.

### HiSeq 2500 mRNA-Seq

Libraries were prepared from total RNA samples using TruSeq Stranded mRNA Sample Preparation Kit followed by cBot cluster generation and 2×100 cycles paired-end HiSeq sequencing. Data mapping was done using Tophat v.2.0.9; transcript assembly was performed by Cufflinks v.2.1.1 followed by differential expression and statistical testing using Cuffdiff v.2.1.1. Log2 fold change was used as a cut-off with FDR adjusted (q) *p* < 0.05 being significant. Heat maps were generated using JMP v.11 software.

### Statistical analysis

All experiments were performed in at least triplicates to calculate means and SEM. One-way ANOVA followed by paired Student's *t-*test were used to assess the differences between samples, unless otherwise specified. *P* < 0.05 was considered significant. All statistical tests are run against normal DBA source unless indicated otherwise in the text or figure legends.

For a detailed description of relevant methods see Supplemental Methods.

## SUPPLEMENTARY MATERIALS AND TABLES


