# Supplementary material for: Role of mir-15a/16-1 in early B cell development in a mouse model of chronic lymphocytic leukemia
Source: Oncotarget. 2016 Aug 14;7(38):60986–99. doi: 10.18632/oncotarget.11290 (PMC5308631; doi:10.18632/oncotarget.11290)
Supplement: Supplementary file 1 [file oncotarget-07-60986-s001.pdf]

Role of mir-15a/16-1 in early B cell development in a mouse model of chronic lymphocytic leukemia

Supplementary Material

Methods

Cell Lines: NZB derived malignant B-1 cell line LNC was used as an *in vitro* model of murine CLL[19] . As a non-CLL B cell line, BALB/c B cell lymphoma A20 control cell line (ATCC #TIB-208) was used. All cell lines were maintained in RPMI 1640 medium supplemented with 10% fetal bovine serum, 1% sodium pyruvate, 1% penicillin–streptomycin at 37°C and 5% CO2. HEK293 cell line was used for lentiviral production. This was maintained in DMEM supplemented with 10% FBS, 1% sodium pyruvate, 1% penicillin–streptomycin at 37°C and 5% CO2. For *in vitro* differentiation of primitive hematopoietic stem cells with the phenotype (lineage$^-$, Sca1$^+$ c-Kit$^+$) or LSK progenitors, the OP9 (ATCC® CRL-2749) stromal cell line was used. Alpha Minimum Essential Medium without ribonucleosides and deoxyribonucleosides and with 2.2 g/L sodium bicarbonate with fetal bovine serum to a final concentration of 20% was used for OP9 system co-culture. NZB ES cells were a kind gift from Dr. Ken-Ichi Yagami (Laboratory Animal Resource Center, University of Tsukuba, Tsukuba, Ibaraki Japan). Wild type (C57BL/6J) ES cells were provided by Dr. Diego Fraidenraich (Department of Cell Biology and Molecular Medicine, Rutgers University, Newark, NJ). All ES and iPS cells were maintained on mitomycin treated mouse embryonic fibroblasts (MEF, Millipore, Danvers, MA) in ES cells medium consisting of Dulbecco's modified Eagle's medium supplemented with 1x nonessential amino acids, 1x penicillin– streptomycin, 1x L-glutamine (all from Life Technologies, Grand Island, NY), 15% knockout serum replacement (Gibco, Life Technologies, Grand Island, NY), 2-

mercaptoethanol (Sigma-Aldrich, St. Louis, MO), and leukemia inhibitory factor (Millipore, Danvers, MA).

Mice: NZB/BlNJ (stock #000684) and DBA/2J (wild type control strain; stock #000671) mice were purchased from Jackson Laboratory (Bar Harbor, ME, USA) and housed under standard pathogen-free conditions at the research animal facility at Rutgers University—New Jersey Medical School, Newark, NJ, USA. DBA congenic strain bearing NZB *mir-15a/16-1* loci (DBA$^{-/-}$) were generated by systematic back-crossing as described previously [20,43]. The immunodeficient NOD.Cg-*Prkdc*^{scid} *Il2rg*^{tm1Wjl}/SzJ (NSG) mice were purchased from Jackson Laboratory (www.jax.org) and housed under standard pathogen free conditions. Mouse femurs and tibias were flushed to harvest cells from the bone marrow as previously described[44]. Twenty thousand HCS or B1P were sorted from the bone marrow source and used for retro-orbital intravenous adoptive transfer experiments (in 200 uL of sterile PBS). NSG recipients were sub-lethally irradiated by 275 Gr prior to injection of sorted cells. Mice were sacrificed 48 days post injection followed by harvesting of cells and flow cytometry analysis. All studies were in compliance with principles of laboratory animal care guidelines and were IACUC approved.

Flow Cytometry and Cell Sorting: For identification of malignant B-1 cells, single-cell suspensions were made from spleen, bone marrow and peritoneal cavity (PerC) cells (referred to as peritoneal washout cells – PWC) and surface stained with anti-mouse IgM (RMM-1, PE-Cy7, Biolegend) and anti-mouse CD5 (APC, BD Pharmingen), CD45R (B220)-Pacific Blue (BD Pharmingen), CD19-APC-Cy7 (1DR, BD Pharmingen), CD11b-AlexaFluor700 (M1/70, Biolegend), CD3-PE (17A2, BD Pharmingen), IgD-PerCP-Cy5.5 (11-26c.2a, Biolegend) and CD138-PE (281-2, BD Pharmingen). Phenotypically B1 cells were defined as CD3^{-} CD19^{+}CD5^{dull}B220^{dull} population whereas B2 cells were CD3^{-}CD19^{+}CD5^{-}B220^{hi}. At least 30,000 events were acquired on a LSR II, and data were analyzed using FlowJo software (Tree Star, Inc., Ashland OR). Antibody cocktail was prepared in 150ul 1X Sorter buffer per tube (0.8ul of

each antibody per tube) and cells were incubated for 20min at 4°C, washed twice with cold sorter buffer and re-suspended in 350 ul sorter buffer (if acquired immediately) or 350ul 2% paraformaldehyde. Acquisition and analysis were performed using FACSDiva Software 6.0 (BD Biosciences).

For B1 progenitors staining and sorting lineage cocktail PE conjugated anti-mouse Ter119 (BD Pharmingen), NK1.1 (PK136, BD Pharmingen), CD3 (17A2, BD Pharmingen), CD4 (GK1.5, BD Pharmingen), IgM (R6-602, BD Pharmingen), Gr1 (RB6-8C5, BD Pharmingen) and CD93-APC (AA.4.1, Biolegend), B220-PacificBlue (BD Pharmingen), CD19-APC-Cy7 (1DR, BD Pharmingen) anti-mouse antibodies were used. B1 progenitors (B1P) were defined as Lineage^{-} AA4.1^{+}CD19^{+}B220$^{neg/dull}$ population. For HSC sorting the same lineage cocktail with the addition of B220-PE (RA3-6B2, 1DR, BD Pharmingen) and c-kit-PerCP-Cy5.5, Sca1-PECy-7, CD105-APC antibodies were used. The HSC population phenotype was Lineage^{-}CD105^{+}Sca-1^{+}c-kit^{+}. LSK progenitors were sorted based on Lin^{-} (anti-mouse Ter119, NK1.1, CD3, CD4, IgM, Gr1, B220), c-kit^{+} (PerCP-Cy5.5), Sca1^{+} (PECy-7) phenotype.

iPS cells generation and lentiviral constructs: For iPS production, the packaging (psPAX2, Addgene #12260) and envelope (pMD2.G, Addgene, #12259) plasmids along with polycistronic lentiviral construct (pKP332, Addgene #21627) were mixed in 4**:**3.5**:**7.5 ratio and transfected using Lipofectamine-2000 (Invitrogen, Carlsbad, CA) following manufacturer's instructions into 293T. The pseudoviral particles were concentrated from supernatant by centrifugation at 26,000 rpm for 90 minutes at 8°C in an SW-28 rotor using a Beckman XL-100 ultracentrifuge and viral titer was confirmed using LentiX GoStix assay (Clontech). For iPS cell induction, 3×10^5 NZB mouse splenic stromal cells (NSF) were seeded onto one well of a six-well plate. The next day, 2.5 uL of the concentrated virus was mixed with 2 ml of ES cell medium containing 8 µg/ml polybrene and added to the target cells. Forty-eight hours later, the NSFs were trypsinized and transferred to a 100-mm dish without MEFs and continuously cultured on the same dish for 3

weeks with daily media changes. ES media with LIF and differentiation inhibitors (2i) was used. Potential iPS cell colonies started to appear after 2–3 weeks. These colonies were individually picked and expanded on MEFs for analysis and subsequent experiment. One million iPS cells in 100 uL of PBS were injected via a 21-G needle into the dorsal flanks of immunodeficient (NOD/SCID) mice subcutaneously. Teratomas were recovered 4–5 weeks post-injection and processed for histological H&E staining and analysis. Photographs were taken and analyzed by an expert pathologist for the presence of the tissues derived from three different germinal layers – ectoderm, mesoderm and endoderm. For alkaline phosphatase staining, 100 to 200 iPS cells were seeded onto one well of a six-well plate with pre-seeded mouse embryonic fibroblasts (MEF) and cultured for one week with daily ES media changes. iPS cells were then stained using the Vector Blue Alkaline Phosphatase Substrate Kit III according to the manufacturer's instructions.

For LNC transduction, the miR-15a-lenti-GFP (SBI, Mountain View, CA) lentiviral aliquot was thawed on ice and added dropwise to the pre-seeded (24 h) culture plates (MOI 10). Polybrene was added at a final concentration of 8 ug/mL to increase transduction efficiency. The plates were incubated at 32°C for 24 hours. Lenti-GFP lentivirus was used as a negative control. For NZB cell line transduction LNC cells were plated 24 hours before at a concentration of $0.3x10^6$/mL in a 6 well plate. Concentrated lentiviral aliquot was thawed on ice and added dropwise to the culture plates (MOI 10). Polybrene was added at a final concentration of 8 ug/mL to increase transduction efficiency. The plates were incubated at 32°C for 24 hours instead of 37°C to assure the virus stability. The transduction efficiency was tested qualitatively using fluorescence microscopy followed by quantitative assessment by flow cytometry. GFP lentivirus (with no miR-15a/16-1) was used as a negative control for miR-15a overexpression.

Quantitative PCR: Total RNA was isolated from cells with Trizol reagent (Invitrogen). RNA was pretreated with RQ1 RNase-free DNase (Promega, Madison, WI) and reverse transcribed with

SuperScript First-Strand Synthesis System (Invitrogen) using oligo d(T)n. Gene expression levels were measured using Power SYBR Green Kit (Ambion Inc., Austin, TX, USA) or SensiMix SYBR Low-ROX Kit (Bioline, London, UK) and custom designed primers (**Supplemental Table 2**). MicroRNA-specific cDNA was prepared using the TaqMan MicroRNA Reverse Transcription Kit (Applied Biosystems) according to the manufacturer's instructions. The qPCR reaction was performed on 7500 Real-Time PCR System for 40 cycles at 60°C. The standard $2^{-\Delta\Delta CT}$ method was used for relative quantitation of microRNA levels using AB7500 software (v. 2.0.6, Applied Biosystems). The following pre-made TaqMan Assays (Life Technologies) were used for real-time quantitation—mmu-miR-15a (assay ID 000389), mmu-miR-150 (assay ID002637) and U6 (Assay ID001973, housekeeping control).

RNA FISH: Dleu2, PU.1, IL10 and Pax5 RNAs were imaged using single-molecule RNA FISH custom probes as previously described [22]. Briefly, a set of 35 probes was designed to hybridize to each target RNA and was synthesized with a 3′ amino modification from Biosearch Technologies (Novato, CA, USA). The individual probes for a given target were pooled in equimolar amounts and then coupled with succinimidyl ester of TMR (for *Dleu2 and IL10*), Alexa594 (for *Pax5*) or Cy5 (for PU.1). The coupled fraction was purified using high-performance liquid chromatography and the concentration was determined using Nanodrop. The coverslips were washed with 1 × phosphate-buffered saline, fixed in 4% formaldehyde, permeabilized with 70% ethanol and hybridized with the *chosen* probes. Hybridization was carried out overnight at 37 °C. The coverslips were washed (with 10% formamide in 2 × SSC) to remove unbound probes and imaged using Zeiss wide field fluorescence microscope (Carl Zeiss, Thornwood, NY, USA). For each image, z-stacks were obtained and merged to get the final image. The image acquisition was carried out by Openlab software (Perkin-Elmer, Waltham, MA, USA) and numbers of mRNAs were counted using custom written algorithms in MATLAB (MathWorks, Natick, MA, USA). LNC cell line was analyzed using individual probes

separately whereas primary splenocytes were probed for three target mRNAs simultaneously to count $Pax5^+$ B cells only.

In vitro Differentiation: OP9 cells were used for iPS or ES *in vitro* differentiation according to published protocols with slight modifications[23]. To initiate co-culture, ES or iPS cells were harvested as a single cell suspension by trypsin-mediated disaggregation and added to OP9 monolayer, with changes of media with culture. The non-adherent cells were reseeded on fresh OP9 and 5 ng/mL of recombinant human Flt-3L was added and cultured with media changes. The non-adherent cells were seeded in 3 mL per well of a 6-well plate containing OP9 cells to generate B-lineage and myelo-erythroid cells. The cytokines were added to each well at final concentrations of 5 ng/mL for Flt-3L and 1 ng/mL for IL7. On day 12 the cells were replated on fresh OP9 with 5 ng/mL Flt-3L and 1 ng/mL IL-7 and the media changed at interval. At day 38 lymphocyte-like non-adherent cells were harvested and analyzed by flow cytometry.

For LSK differentiation, bone marrow cells from age-matched DBA, NZB and $DBA^{-/-}$ congenic mice femurs and tibiae were obtained. Using flow cytometric cell sorting, LSK population was isolated based on $CD117^+$ $Sca\text{-}1^+$ $Lineage^{neg}$ (CD4, CD8, CD19, CD45R, GR.1, Ter119) profile. 2×10^4 sorted LSK cells were plated with OP9 cells with 5 ng/mL Flt-3L and 1 ng/mL IL-7 and feed and reseeded. At day 11 the cells were analyzed by flow cytometry using B cell specific antibodies.

Supplemental Tables

Table S1. List of antibodies for flow cytometry, sorting, immunofluorescence and immunoblotting.

Target antigen	Host	Specificity	Clone	Conjugated fluorophore	Manufacturer
IgM	Rat	Mouse	RMM-1	PE-Cy7	Biolegend
CD5	Rat	Mouse	53-7.3	APC	BD Pharmingen
CD45R (B220)	Rat	Mouse	RA3-6B2	Pacific Blue	BD Pharmingen
CD19	Rat	Mouse	1DR	APC-Cy7	BD Pharmingen
CD11b	Rat	Mouse/ Human	M1/70	Alexa Fluor 700	Biolegend
CD3	Rat	Mouse	17A2	PE	BD Pharmingen
IgD	Rat	Mouse	11-26c.2a	PerCP-Cy5.5	Biolegend
CD138	Rat	Mouse	281-2	PE	BD Pharmingen
Ter119	Rat	Mouse	TER-119	PE	BD Pharmingen
NK1.1	Mouse	Mouse	PK136	PE	BD Pharmingen
CD4	Rat	Mouse	GK1.5	PE	BD Pharmingen
IgM	Rat	Mouse	R6-602	PE	BD Pharmingen
Gr1	Rat	Mouse	RB6-8C5	PE	BD Pharmingen
AA4.1 (CD93)	Rat	Mouse		APC	Biolegend
CD45R (B220)	Rat	Mouse	RA3-6B2	PE	BD Pharmingen
c-kit	Rat	Mouse	2B8	PerCP-Cy5.5	BD Pharmingen
Sca-1	Rat	Mouse	D7	PE-Cy7	BD Pharmingen
CD105	Rat	Mouse	MJ7/18	APC	Biolegend
Mmp10	Rabbit	Human mouse, rat	C0263	unconjugated	Sydlabs
Pax-5	Rat	Mouse, human	1H9	unconjugated	Biolegend
PU.1	Rat	Mouse	823123	unconjugated	R&D Systems
cMyb	Rabbit	Mouse	M-19	unconjugated	Santa Cruz Biotechnology
b-actin	Mouse	Mouse, human, rat	AC-15	unconjugated	
Nanog	Rabbit	Mouse, human	N/A	unconjugated	Stemgent
SSEA-1	Mouse	Human, mouse, rat	MC-480	PE	Millipore
Secondary IgG	Goat	Rabbit	111-116-144	PE	Jackson Immunoresearch Laboratories
H-2D[d]	Mouse	Mouse	N/A	FITC	BD Pharmingen

Table S2. List of primers for PCR and qPCR analysis

No.	Primer set	Forward	Reverse
1	Pax5	ACCATCAGGACAGGACATGG	TTGGCGTTTGTACTCAGCGA
2	β-actin	AGATCAAGATCATTGCTCCTCC	TCAAAGAAAGGGTGTAAAACGC
5	Nanog	GACTGTGTTCTCTCAGGCCC	CTGGTGCTGAGCCCTTCTGA
6	CD11b	GTCAGTGTGTCCCTTGCCTC	CTATGATCCGCTGGCTGTGG
7	OSK-TG	GATGAACTGACCAGGCACTA	GATTATCGGAATTCCCTCGAG
8	Oct-4	AGCTTGGGCTAGAGAAGGAT	TCAGTTTGAATGCATGGGAG
9	Sox-2	TGCACATGGCCCAGCACTA	TTCTCCAGTTCGCAGTCCAG
10	cMyc	GTTGGAAACCCCGCAGACAG	CGACCGCAACATAGGATGGA
11	PU.1	CTCACCCAGGGCTCCTGTAG	CCAAGTCATCCGATGGAGGG

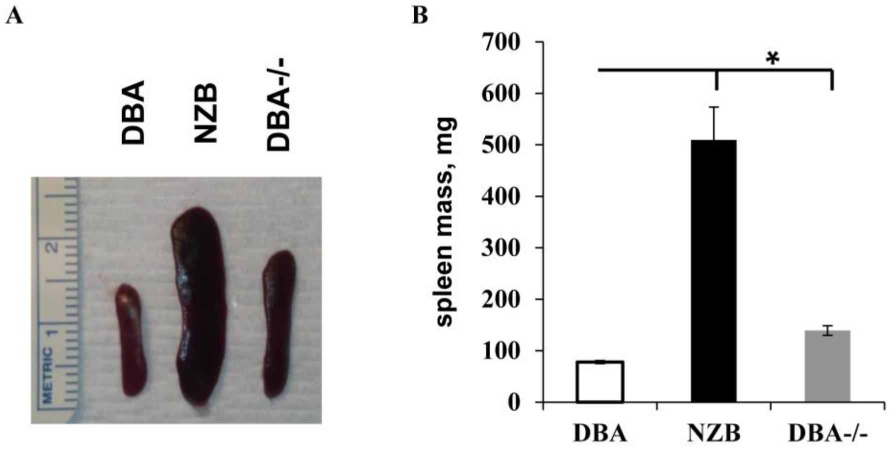

Figure S1. DBA, NZB and DBA$^{-/-}$ congenic mice splenic phenotype: (A) Spleens from age-matched DBA, NZB and congenic DBA$^{-/-}$ mice. (B) Comparison of spleen sizes of control DBA, NZB and DBA$^{-/-}$ animals (in milligrams). Mice were 15 months of age, with $n \geq 3$. Columns represent means and bars represent SEM. $P<0.05$ was considered significant (marked with an asterisk).

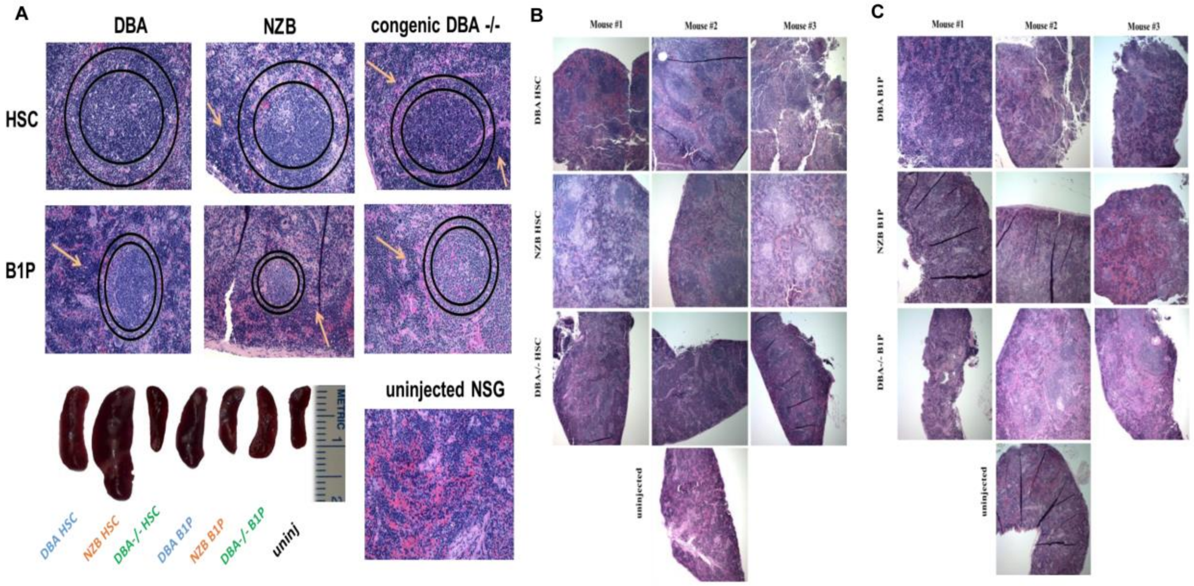

Figure S2. Histological Analysis of the spleens of NSG recipient animals following transfer of either HSC or B1P sources derived from wild-type DBA, NZB or DBA -/- congenic mice: (A) Spleens from NSG recipients were photographed (bottom left) and then sectioned for H&E staining (20x). Circles represent germinal centers and mantle zones, arrows indicate clusters of lymphoid cells in the marginal zone areas. The uninjected NSG spleen is shown as a control (note the absence of any germinal centers); (B) Histological H&E staining of the NSG recipients' spleens (10x) after DBA, NZB and $DBA^{-/-}$ HSC engraftment at day 48; (C) Histological H&E staining of the NSG recipients' spleens (10x) after DBA, NZB and $DBA^{-/-}$ B1P engraftment at day 48.

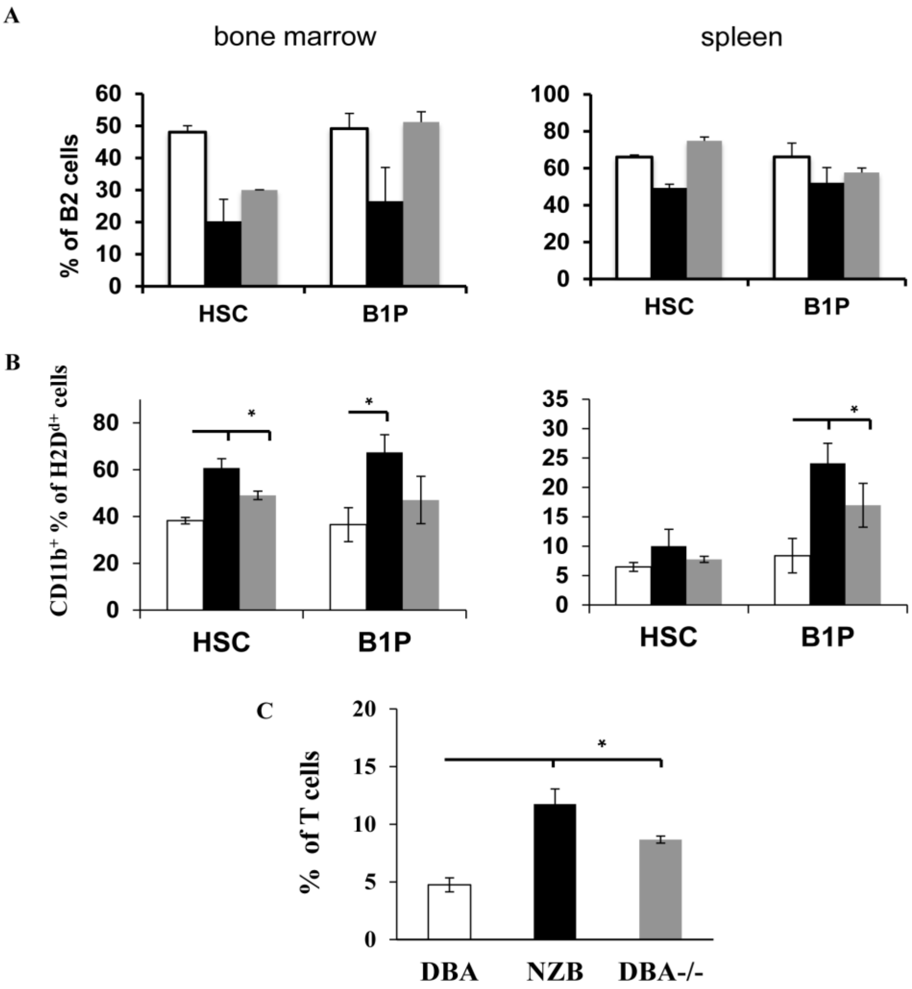

Figure S3. Quantitative analysis of B2 cells, myeloid lineage cells and T cells in the spleens of NSG recipients: (A) The percentage of $CD5^{-}B220^{bright}$ B2 cells derived from donor ($H2D^{d+}$) cells in recipients' bone marrow (left) and spleen (right); (B) $CD11b^{+}$ population quantification in bone marrow (left) and spleen (right) of NSG recipients; (C) T cells counts (%) in HSC transplanted NSG recipients' spleens. N=3, columns are means and bars are SEMs. $P<0.05$ was considered significant (marked with an asterisk).

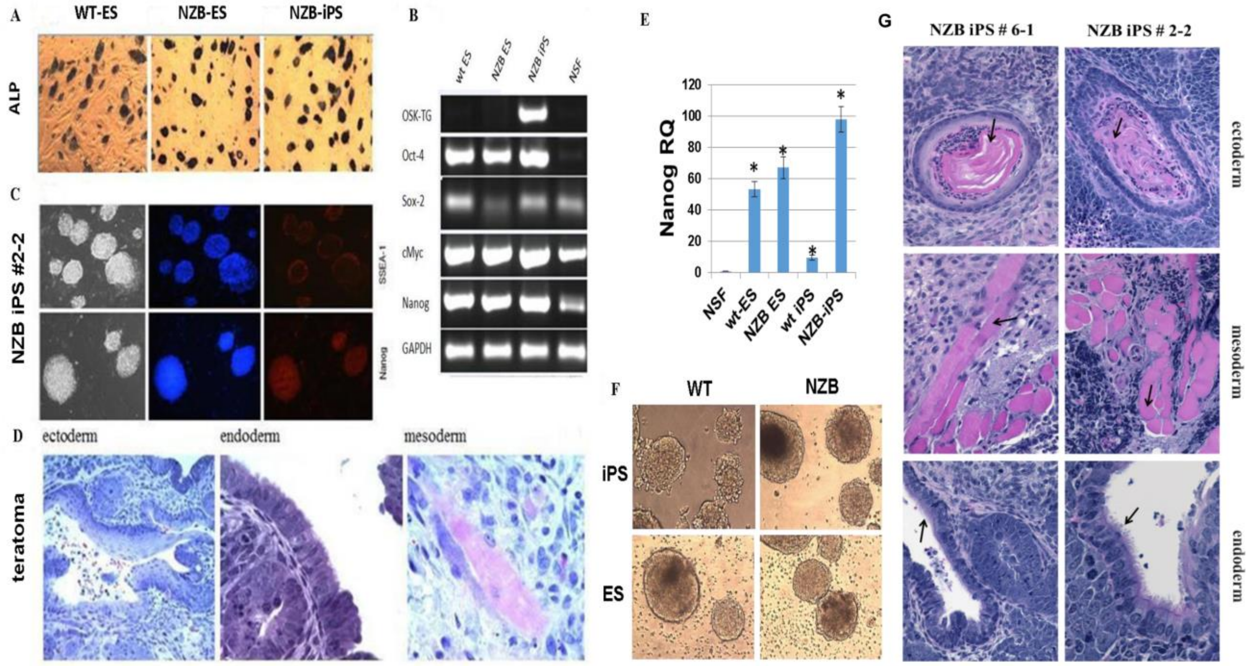

Figure S4. NZB iPS cells pluripotency analysis: Culture of ES cells from NZB and wild-type mice (C57BL/6J), NZB iPS colonies and NZB spleen cell fibroblasts (NSF) were analyzed. (A) Alkaline phosphatase staining of NZB iPS cells. C57BL/6 ES (WT) and NZB ES cells were used as control. (B) Routine PCR analysis of polycistronic OSK transgene (OSK-TG) and endogenous Oct4, Sox2, Klf4, cMyc and Nanog genes expression in generated NZB iPS cells. WT-ES and NZB ES cells were used as positive control; NZB spleen stromal cells were used as negative control. GAPDH expression was used as endogenous control. (C) Immunofluorescent analysis of NZB iPS cell colonies for Nanog (intracellular) and SSEA-1 (surface). Bright field (left), DAPI counterstaining (middle) and PE-conjugated antibody staining (right) is shown. (D) Teratoma formation assay for NZB iPS cell clone #1-1 injected into NOD-SCID mouse for 4.5 weeks. (E) qPCR analysis of Nanog levels in ES and iPS cells compared to the non-pluripotent fibroblast (NSF). (F) WT and NZB ES/iPS cells pre-differentiated on low adherence plates for 7 days. (G) Additional teratoma formation assays on NZB iPS cells. $2x10^6$ NZB iPS #1-1 cells were injected into dorsal flanks of NOD/SCID mice subcutaneously. Four to five weeks later the mice were sacrificed and teratomas were harvested, minced and processed for H&E staining. Tissues derived from ectoderm (keratinized epithelium), mesoderm (striated muscles) and endoderm (ciliated epithelium) are shown (indicated by arrows).

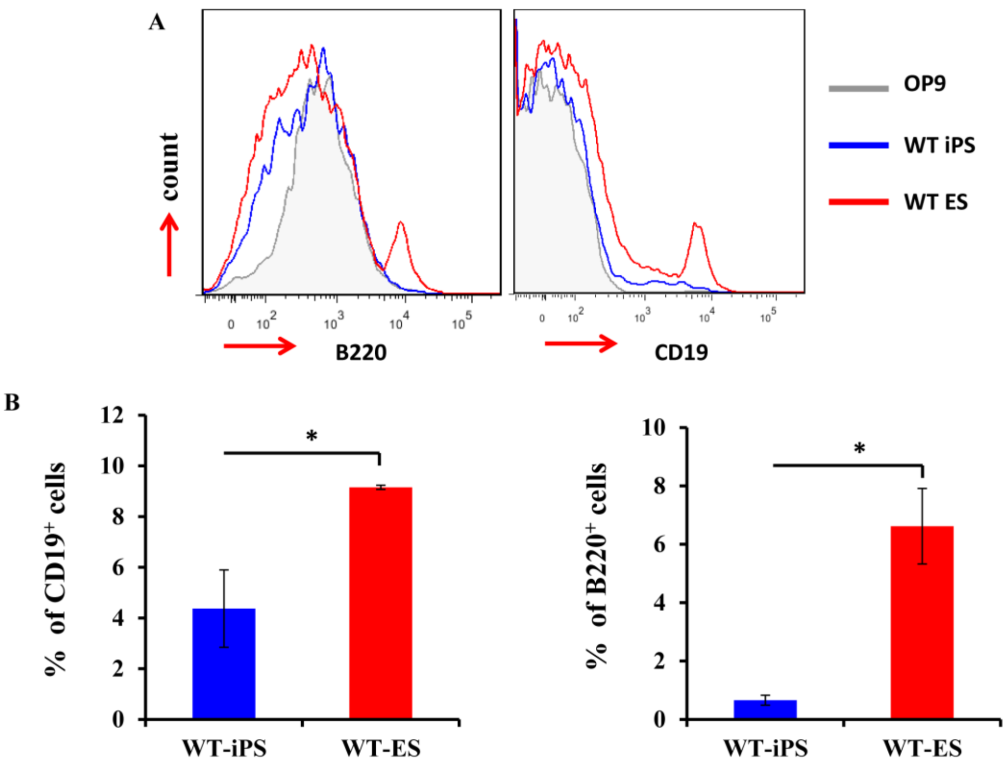

Figure S5. *In vitro* differentiation of pluripotent stem cells: (A) Flow cytometry histogram of WT (C57BL/6J) iPS and ES cells co-cultured with OP9 stromal cells in medium supplemented with IL7 and Flt3L, day 38. B cell B220 and CD19 surface markers expression is shown. OP9 feeder layer cells were used as control. (B) Quantitative analysis of B lineage cells derived from WT iPS and ES cells *in vitro*. Percentages of CD19+ cells (left) and B220+ (right) are displayed. N=3, columns represent percentage of all live cells, bars are SEM. * P <0.05 is considered significant.

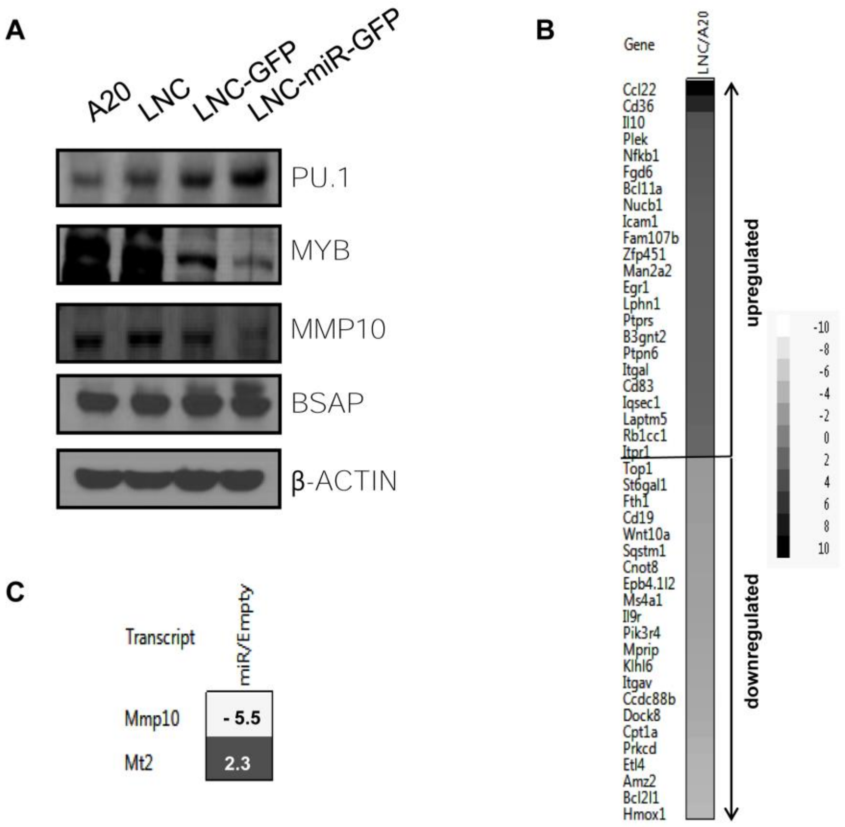

Figure S6. Illumina HiSeq2500 RNA-SEQ and immunoblotting analysis of LNC cells transduced with lentiviral miR-15a construct: (A) Heatmap diagram for significantly up- and downregulated transcripts in LNC cells relative to A20 control cell line. (B) Gene transcripts significantly downregulated (Mmp10) and upregulated (Mt2) following exogenous delivery of miR-15a; q<0.05 (FDR adjusted p value) was considered significant. (C) Western blot analysis of LNC cell line with and without transduction with lentiviral miR-15a construct. The A20 cell line is a control non-NZB B cell line. β-actin was used as a housekeeping control.
